# Reconhecimento pelo Médico Regulador das Expressões Usadas por Leigos ao Pedirem Ajuda em Suposta Parada Cardiorrespiratória

**DOI:** 10.36660/abc.20240669

**Published:** 2024-11-27

**Authors:** Ronaldo C. O. Vinagre

**Affiliations:** 1 Universidade Federal do Rio de Janeiro Rio de Janeiro RJ Brasil Universidade Federal do Rio de Janeiro, Rio de Janeiro, RJ – Brasil

**Keywords:** Parada Cardíaca Extra-Hospitalar, Assistência Pré-hospitalar, Operador de Emergência Médica

A parada cardíaca fora do hospital (PCFH) é uma emergência com risco de vida e com tempo limitado que ocorre milhões de vezes por ano. Dados de países ao redor do mundo com serviços médicos de emergência (SME) em vigor sugerem uma média global de 82,1 PCFHs atendidos por SME por 100.000 pessoas por ano. A sobrevivência após a PCFH permanece modesta, apesar dos protocolos de despacho padronizados em sistemas de SME, do aumento do treinamento comunitário e da introdução de cuidados pós-ressuscitação. Dez por cento (variação, 6%–22%) das pessoas que sofrem PCFHs podem esperar sobreviver com um resultado neurológico favorável. No entanto, as intervenções pré-hospitalares precoces têm um impacto substancial na sobrevivência das vítimas de PCFH.^1^

A reanimação cardiopulmonar (RCP) iniciada por um observador aumenta as chances de sobrevivência em 30 dias em duas vezes e está associada a melhores resultados neurológicos em longo prazo. Portanto, o reconhecimento precoce da parada cardíaca é a pedra angular da cadeia de sobrevivência. Sinais clínicos bem conhecidos de parada cardíaca são falta de resposta e respiração ausente ou anormal. No entanto, não está claro como esses sinais e sintomas, especialmente respirações agonísticas, são interpretados e descritos por leigos. Além disso, a RCP assistida por despachante aumenta a sobrevivência neurologicamente intacta em PCFH, de acordo com vários estudos.^1,2^

Chamadas de emergência podem conter palavras-gatilho hipotéticas que o protocolo de despacho atual pode não reconhecer; o *International Liaison Committee for Resuscitation* (ILCOR) anunciou que palavras-gatilho constituem uma lacuna de conhecimento científico. Essas palavras-gatilho podem ser usadas para facilitar o reconhecimento de parada cardíaca, reduzir o tempo de despacho do SME e aumentar as taxas de RCP imediatas de observadores. Mais importante, elas podem ser usadas para reduzir o número de alarmes falso-positivos e, assim, melhorar a especificidade do reconhecimento de parada cardíaca.^3^

O Estudo Estudo Observacional de Expressões Ditas por Solicitantes de Atendimento Emergencial para uma Parada Cardiorrespiratória e o Impacto no Reconhecimento pelo Médico Regulador ^4^ é o primeiro estudo realizado no Brasil que analisa gravações em áudio de chamadas de socorro em parada cardíaca (PCR), avaliando categorias e subcategorias de palavras/expressões que influenciam no reconhecimento dessa condição pelo médico regulador, que é o primeiro a fazer contato com o chamador no Serviço de Atendimento Móvel de Urgência (SAMU). O estudo^4^ destaca que, no futuro, combinações de palavras-chave/expressões poderão ser utilizadas para estabelecer rotinas que visem melhorar a qualidade da compreensão precoce da PCR pelos médicos reguladores do SAMU. Os resultados deste estudo^4^ também apontam para a necessidade de melhorar o processo de comunicação entre pessoas da comunidade e médicos reguladores para aumentar gradativamente o reconhecimento real da situação descrita e, a partir desse conhecimento, oferecer RCP eficaz e precoce em uma situação de PCR. Já existem sistemas de reconhecimento automático de fala que demonstraram melhor assertividade do que os médicos reguladores. O estudo acrescenta que, portanto, pode-se considerar que uma associação de termos utilizados pelos chamadores por meio do reconhecimento automático de fala associado ao aprendizado de máquina poderia trazer uma perspectiva de resposta em larga escala aos chamados de emergências médicas.^4^ Além disso, o conhecimento gradual e evolutivo dos fatores de confusão para o reconhecimento de PCR pode auxiliar na produção de protocolos de atendimento e estratégias de treinamento mais eficazes para o médico regulador, melhorando e qualificando cada vez mais a comunicação com o chamador leigo. Com o avanço do conhecimento e aprimoramento do conhecimento e compreensão das expressões facilitadoras, estas podem ser incorporadas rotineiramente aos protocolos de regulação de casos suspeitos de PCR.

Portanto, este é o primeiro estudo^4^ realizado no Brasil, com dados brasileiros, sobre a estrutura de atendimento de emergência oferecida pelo SAMU, com pessoas da comunidade atuando e sendo atendidas pelos médicos reguladores, em uma atividade que traz à tona, inicialmente, a realidade a ser enfrentada com o estabelecimento de ações que visem melhorar a compreensão de palavras e expressões utilizadas por pessoas da comunidade que, na maioria dos casos no Brasil, ainda não têm o conhecimento necessário para informar adequadamente ao médico regulador a real situação daquele momento, utilizando, na maioria dos casos, palavras e expressões cotidianas que acabam confundindo e não refletem a RCP já instalada para este médico.

Este é um estudo^4^ de extrema relevância, e deve haver desdobramentos para que se crie uma rede de estudos e, a partir daí, protocolos e ações que possam contribuir para melhorar os índices de eficiência na RCP e, principalmente, para uma evolução mais efetiva e favorável das pessoas com RCP atendidas por leigos no Brasil.
