# Supplementary material for: Reconhecimento pelo Médico Regulador das Expressões Usadas por Leigos ao Pedirem Ajuda em Suposta Parada Cardiorrespiratória
Source: Arq Bras Cardiol. 2024 Nov 27;121(11):e20240669. [Article in Portuguese] doi: 10.36660/abc.20240669 (PMC11634313; doi:10.36660/abc.20240669)
Supplement: Translation

# Translation: Recognition by the Regulatory Physician of Expressions Used by Lay People when Asking for Help in Supposed Cardiorespiratory Arrest

**DOI:** 10.36660/abc.20240669i

**Authors:** Ronaldo C. O. Vinagre

**Affiliations:** 1 Rio de Janeiro RJ Brazil Universidade Federal do Rio de Janeiro, Rio de Janeiro, RJ – Brazil

**Keywords:** Out-of-Hospital Cardiac Arrest, Prehospital Care, Emergency Medical Dispatcher

Out-of-hospital cardiac arrest (OHCA) is a time-sensitive, life-threatening emergency that occurs millions of times every year. Data from countries around the world with emergency medical services (EMS) in place suggest a global average of 82.1 EMS-attended OHCAs per 100,000 people per annum. Survival after OHA remains modest despite standardized dispatch protocols in EMS systems, increased community training, and the introduction of post-resuscitation care. Ten percent (range, 6%–22%) of people who experience OHCA can expect to survive with a favorable neurological outcome. However, early prehospital interventions have a substantial impact on the survival of OHA victims.^1^

Bystander-initiated cardiopulmonary resuscitation (CPR) increases the odds of 30-day survival by two-fold and is associated with improved long-term neurological outcomes. So, early recognition of cardiac arrest is the cornerstone of the chain of survival. Well-known clinical signs of cardiac arrest are unresponsiveness and absent or abnormal breathing. However, it is unclear how these signs and symptoms, especially agonistic respirations, are interpreted and described by laypeople. Besides that, dispatcher-assisted CPR increases neurologically intact survival in OHCA, according to several studies.^1,2^

Emergency calls may contain hypothetical trigger words that current dispatch protocol may not recognize; the International Liaison Committee for Resuscitation (ILCOR) has announced that trigger words constitute a scientific knowledge gap. These trigger words can be used to facilitate the recognition of cardiac arrest, to reduce EMS dispatch time, and to increase immediate bystander CPR rates. Importantly, they can be used to reduce the number of false-positive alarms and thus improve the specificity of cardiac arrest recognition.^3^

The Observational Study of Words Used by Emergency Callers and Their Impact on the Recognition of an Out-Of-Hospital Cardiopulmonary Arrest by the Medical Dispatcher^4^ is the first study conducted in Brazil that analyzes audio recordings of calls for help in cardiac arrest (CA), evaluating categories and subcategories of words/expressions that influence the recognition of this condition by the regulatory physician, who is the first to make contact with the caller at the Mobile Emergency Care Service (SAMU). The study^4^ highlights the fact that in the future, combinations of keywords/expressions could be used to establish routines aimed at improving the quality of early understanding of CA by SAMU regulatory physicians. The results of this study^4^ also point to the need to improve the communication process between people in the community and regulatory physicians to gradually increase the real recognition of the situation described and, based on this knowledge, offer effective and early CPR in a CA situation. There are already automatic speech recognition systems that have demonstrated better assertiveness than regulatory physicians. The study adds that, therefore, it can be considered that an association of terms used by callers through automatic speech recognition associated with machine learning could bring a perspective of large-scale response to calls for medical emergencies.^4^ Furthermore, the gradual and evolving knowledge of the confounding factors for the recognition of CA can assist in the production of more effective care protocols and training strategies for the regulating physician, increasingly improving and qualifying communication with the lay caller. With the advancement of knowledge and improvement of the knowledge and understanding of facilitating expressions, these can be routinely incorporated into the regulation protocols for suspected cases of CA.

Therefore, this is the first study^4^ conducted in Brazil, using Brazilian data, the emergency care structure offered by SAMU, with people from the community working and being responded to by medical regulators, in an activity that sheds initial light on the reality to be faced with the establishment of actions aimed at improving the comprehension of words and expressions used by people in the community who, in most cases in Brazil, still do not have the necessary knowledge to adequately inform the regulatory doctor of the real situation at that moment, using in most cases everyday words and expressions that end up confusing and do not reflect the CPR already installed for this doctor.

This is an extremely relevant study, and there must be developments so that a network of studies can be created and, from there, protocols and actions that can contribute to improving efficiency rates in CPR and, mainly, to achieve a more effective and favorable evolution in people with CPR treated by lay people in Brazil.
